# Pancreatoduodenectomy in a patient with complete atherosclerotic occlusion of superior mesenteric artery: A case report

**DOI:** 10.1016/j.ijscr.2024.110492

**Published:** 2024-10-19

**Authors:** Veronika Rozhkova, Anton Burlaka, Ivan Lisniy, Oleksandr Chukanov, Andrii Beznosenko, Sergii Zemskov

**Affiliations:** a Department of thoraco-abdominal oncology, National Cancer Institute, Kyiv, Ukraine; b Lifescan diagnostic center, Kyiv, Ukraine; c Bogomolets National Medical University, Kyiv, Ukraine

**Keywords:** Superior mesenteric artery occlusion, Pancreatoduodenectomy, Ampullary adenocarcinoma, Case report

## Abstract

**Introduction and importance:**

In elderly patients with atherosclerotic disease the occlusion of splanchnic arteries is a frequently observed variation, which doesn't normally affect patient's condition. There are sporadic reports on pancreatoduodenectomy in these cases.

**Case presentation:**

A 72-year-old female was admitted to our department with cancer of the ampulla of Vater. Pre-operative CT-angiography revealed total atherosclerotic occlusion of the main trunk of the superior mesenteric artery (SMA). Collateral circulation was conducted through gastroduodenal and dorsal pancreatic artery. Pancreatoduodenectomy was performed with an intraoperative clamping test, which showed no signs of bowel ischemia. The patient was discharged on post-operative day 14 without any complications, and long-term follow-up revealed adjustment of collateral circulation through the inferior mesenteric artery and Riolan's arcade.

**Clinical discussion:**

Most cases of mesenteric artery stenosis occur in patients with underlying cardiac condition. As the occlusion develops chronically, it doesn't cause any symptoms due to collateral circulation, and no preoperative intervention is usually needed. However, there are rare cases reported in the literature, when preoperative endovascular stenting and SMA dilation were performed before pancreatoduodenectomy. In this report we decided to proceed with surgery upfront and perform an intraoperative clamping test.

**Conclusion:**

In this report we present a rare case of successful pancreatoduodenectomy in a patient with total atherosclerosis of the superior mesenteric artery. The intraoperative clamping test allowed us to assess both the sufficiency of collateral circulation and the feasibility of the surgery.

## Highlights

• Pancreaticoduodenectomy in patients with a concomitant occlusion of superior mesenteric artery is rare.
• Collateral circulation develops through the inferior mesenteric artery, gastroduodenal artery and dorsal pancreatic artery.
• Intraoperative clamping test allows assessment of the adequacy of blood perfusion and thus safe performance of the surgery.

## 1 Introduction

Chronic mesenteric occlusion is reported in 2–10 % of patients [[1], [2], [3]]. If other vessels are not affected, acute or chronic mesenteric ischemia is less likely to occur due to development of arterial collaterals [4,5]. However, when abdominal intervention is planned, the surgeon must be aware of such anatomical variations. The situation is especially challenging if pancreaticoduodenectomy (PD) is planned and one of the main collateral pathways – gastroduodenal artery and pancreaticoduodenal arcades – must be transected [2]. There are number of cases describing successful pancreatic resection in patients with celiac axis occlusion, but due to lower incidence of the isolated superior mesenteric artery (SMA) stenosis, the data on this is scarce [6].

We describe a case of successful pancreatoduodenectomy in a patient with total occlusion of superior mesenteric artery due to atherosclerosis. Our work has been written according to the SCARE Guidelines 2023 criteria [7].

## 2 Case description

**Fig. 1:**
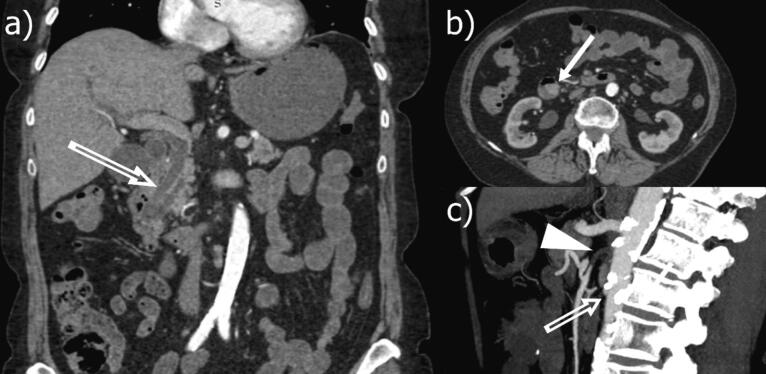
Preoperative CT-reconstruction. a) Double-duct sign shows dilation of bile duct and pancreatic duct (blank arrow). b) Tumor at the ampulla of Vater (filled arrow). c) Total occlusion of the superior mesenteric artery (arrowhead); atherosclerotic plaques at the abdominal aorta (unfilled arrow).

**Fig. 2:**
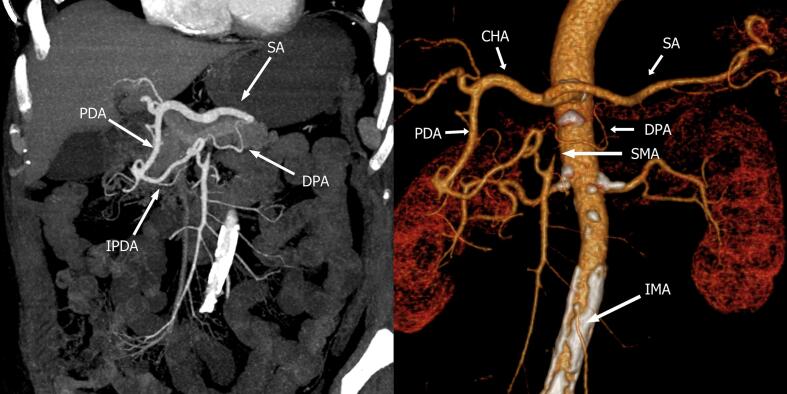
Preoperative CT-reconstruction of arterial blood supply. Shows collateral circulation from celiac axis to SMA through pancreaticoduodenal arcade (PDA) and dorsal pancreatic artery (DPA).

A 72-year-old female was referred to us with jaundice in March 2023. Computer tomography (CT) and subsequent biopsy revealed ampullar adenocarcinoma (Fig. 1). In 2011 the patient underwent total thyroidectomy and was receiving hormonal replacement therapy. Jaundice was treated with sphincterotomy and endoscopic retrograde cholangiopancreatography (ERCP). The patient was presented at a multidisciplinary meeting and a surgical treatment strategy was chosen. Three-dimensional CT showed aortic atherosclerosis with total occlusion of superior mesenteric artery (Fig. 1). We decided to proceed with surgery and intraoperative clamping test, as there was adequate collateral blood flow (Fig. 2).

## 3 Vascular anatomy

CT revealed collateral circulation between the superior mesenteric artery and the celiac artery branches via the gastroduodenal arcade and dorsal pancreatic artery [8] (Fig. 2). The blood supply of the colon was conducted through the inferior mesenteric artery.

CHA: common hepatic artery; CA: Celiac axis; PDA: pancreaticoduodenal arcade; CHA: common hepatic artery; SMA: superior mesenteric artery; SA: splenic artery; IPDA: inferior pancreaticoduodenal artery; IMA: inferior mesenteric artery.

## 4 Surgical technique

We performed a classic Whipple procedure with regional lymphadenectomy. The intraoperatively gastroduodenal artery (GDA) and dorsal pancreatic artery were clamped. No change of color tone in the intestines was observed, the blood pulsation of the marginal branches at the colon was persistent after 45 min of clamping, and therefore the GDA and DPA were ligated. We performed standard single-loop reconstruction and duct-to-mucosa pancreatojejunostomy with internal stenting.

## 5 Outcome

The post-operative course was uneventful. The patient's blood test work-up in pre- and post-operative period is presented in the Table 1. There was an elevation of drainage amylase level > 3 times the upper limit of normal, which was defined as a biochemical leak and which had no clinical impact on the patient's management [9]. Drains were removed on POD (post-operative day) 11, and the patient was discharged on POD 14 without complications.

**Table 1:** Blood test results of the patient. POD: post-operative day.

Timeline	Hemoglobin, g/dL	Amylase, U/L	C-reactive protein, mg/L
Pre-operatively	11,8	20	–
POD 1	10,3	180	73,7
POD 3	97,2	131	65,2
POD 5	93,5	107	49,8
POD 7	10,4	100	35,1

**Fig. 3:**
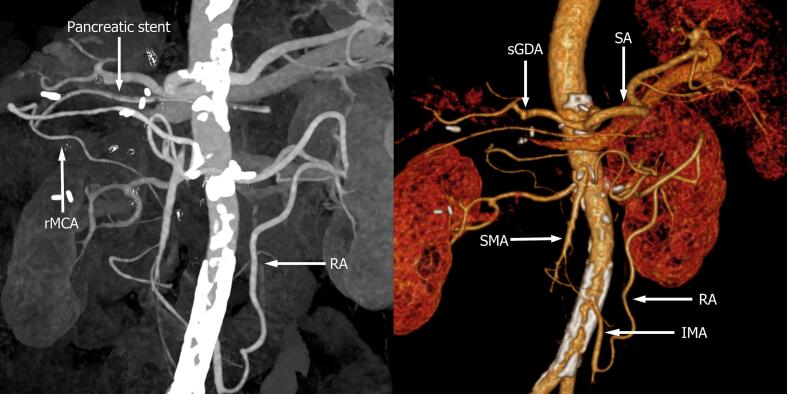
Postoperative CT-reconstruction of arterial blood supply. Development of Riolan's arcade (RA) on POD 32. Dilation of middle colic artery branches. rMCA: right middle colic artery; sGDA: gastroduodenal artery stump;

The postoperative histology revealed mixed type (intestinal and pancreatobiliary) moderately differentiated (G2) ampullary adenocarcinoma. Out of 15 examined lymph nodes, none were metastatic (pT2, pN0 (0/15), M0, G3, LV1, Pn0, R0). Adjuvant chemotherapy was not indicated due to the early stage of the disease. Three-dimensional computer tomography was performed on POD 32 and showed development of Riolan's arcade (Fig. 3).

For the follow-up, the triple-phase CT of the chest, pelvis and abdomen, as well as cancer antigen 19–9 monitoring, were performed at 3, 6 and 14 months after surgery. These tests revealed no evidence of local or distant disease recurrence.

## 6 Discussion

The incidence of total superior mesenteric artery occlusion varies from 2 to 10 % [1]. This condition can become a surgical challenge when the pancreatic resection is planned. Gaujoux et al. (2009) reviewed CT-scans of 545 PD patients, and visceral artery stenosis was detected in 11 % (62) of cases, 5 % (27) were hemodynamically significant [6]. The collaterals, that provide circulation in case of severe stenosis – gastroduodenal artery, pancreaticoduodenal arcades, arch of Büchler, marginal artery of Drummond, and Riolan's arch – need to be taken into consideration [2]. In our case the blood flow was compensated through the enlarged dorsal pancreatic artery, which would have to be sacrificed during pancreaticoduodenectomy. There are two different approaches that may be applied in such cases: either preoperative revascularization, or an intraoperative clamping test. There's no consensus regarding this in the literature, and the data we do have is mostly limited to case-reports from the eastern literature. Similar collaterals as in our case provided circulation for the patient described by Kuise (2023), however, they proceeded with preoperative pancreatic arcade embolization [10]. Consequently, a collateral pathway developed and pancreatoduodenectomy was performed two weeks after. Other authors proceeded with preoperative SMA stenting [4,11]. We decided to perform surgery with an intraoperative clamping test – a tactic that has been chosen by other authors as well [[12], [13], [14]].

Tagkalos et al. (2018) described a case of successful PD in а patient with occlusion of both SMA and CA. Stenosis wasn't detected until POD 3, when transaminases peaked. Heparinization was conducted, however it didn't affect arterial occlusion, and blood supply was provided via IMA branches. Authors noted, that if the specifics of vascular anatomy were diagnosed preoperatively, the bypass procedure would have been performed or surgery would have been denied [13]. Patients with this anatomy, including the presented case, aren't candidates for IMA-compromising surgeries, such as left-hemicolectomy or pancreatectomy combined with bowel resection, unless preoperative revascularization is performed. In the case-series by Gaujoux et al. (2009) one out of the three SMA stenosis was not detected at pre-operative imaging. PD led to mesenteric ischemia and death on POD 2 [6]. Other two cases of SMA occlusion were stented preoperatively.

Celiac artery stenosis has more frequent occurrence among forms of mesenteric ischemia compared to SMA, and it is associated with median arcuate ligament compression or atherosclerosis in elderly patients with coronary artery disease [[15], [16], [17]]. This group of patients is predominant among candidates for pancreaticoduodenectomy, hence, surgeons must be aware of such possibility and pay extra attention to abdominal imaging when PD is planned. CT-angiography provides 96 % sensitivity and 92 % accuracy in detection of arterial stenosis, eliminating the need for invasive angiography [6]. When stenosis of one mesenteric vessel is detected, no revascularization is usually needed [4]. In cases where two and more vessels are impaired, and abdominal intervention is planned, it is recommended to consider endovascular intervention to prevent postoperative mesenteric ischemia. Intraoperative GDA clamping test might be sufficient to estimate the necessity of revascularization, and some authors recommend doing it routinely for all patients undergoing pancreaticoduodenectomy [18,19]. In our case, the GDA clamping test showed no signs of bowel ischemia which allowed us to proceed with surgery.

## 7 Conclusion

We describe a case of successful pancreatoduodenectomy without prior revascularization in a patient with total atherosclerotic occlusion of the superior mesenteric artery. The intraoperative clamping test allowed us to safely perform the surgery. Post-operative arterial collateralization was compensated through Riolan's arcade. It is extremely important to recognize abnormalities in the major abdominal vessels and vascular branches by carefully checking preoperative images before major abdominal surgery.

## Author contribution

Veronika Rozhkova – writing the initial draft and submitting the manuscript.

Anton Burlaka – general idea and editing the manuscript.

Ivan Lisnyy – images design and editing the manuscript.

Oleksandr Chukanov– radiological images design.

Andriy Beznosenko, Sergii Zemskov – supervision and final editing of the manuscript.

All authors took part in final revision and preparation of the manuscript.

## Consent

Written informed consent was obtained from the patient for publication of this case report and accompanying images. A copy of the written consent is available for review by the Editor-in-Chief of this journal on request.

## Ethical approval

Ethical approval for this study (Ethical Committee No 245/5) was provided by the Ethical Committee of National Cancer Institute, Kyiv, Ukraine on 27 March 2024.

## Guarantor

Veronika Rozhkova.

## Funding

This research did not receive any specific grant from funding agencies in the public, commercial, or not-for-profit sectors.

## Declaration of competing interest

Authors declare no competing interests.
